# Tryptophan-2,3-Dioxygenase (TDO) deficiency is associated with subclinical neuroprotection in a mouse model of multiple sclerosis

**DOI:** 10.1038/srep41271

**Published:** 2017-01-24

**Authors:** Tobias V. Lanz, Sarah K. Williams, Aleksandar Stojic, Simeon Iwantscheff, Jana K. Sonner, Carl Grabitz, Simon Becker, Laura-Inés Böhler, Soumya R. Mohapatra, Felix Sahm, Günter Küblbeck, Toshikazu Nakamura, Hiroshi Funakoshi, Christiane A. Opitz, Wolfgang Wick, Ricarda Diem, Michael Platten

**Affiliations:** 1 DKTK Clinical Cooperation Unit Neuroimmunology and Brain Tumor Immunology, German Cancer Research Center (DKFZ), Im Neuenheimer Feld 280, Heidelberg, Germany; 2 Department of Neurology and National Center for Tumor Diseases, University Hospital Heidelberg, Im Neuenheimer Feld 400, 69120 Heidelberg, Germany; 3 AG Neuroinflammation, Department of Neurology, University Hospital Heidelberg, Otto-Meyerhof Zentrum, Im Neuenheimer Feld 350, 69120 Heidelberg, Germany; 4 AG Brain Tumor Metabolism, German Cancer Research Center (DKFZ), Im Neuenheimer Feld 280, Heidelberg, Germany; 5 Department of Neuropathology, University Hospital Heidelberg, and Clinical Cooperation Unit Neuropathology, German Cancer Consortium (DKTK), German cancer Research Center (DKFZ), Im Neuenheimer Feld 224, 69120 Heidelberg, Germany; 6 Department of Molecular Immunology, German Cancer Research Center (DKFZ), Im Neuenheimer Feld 280, Heidelberg, Germany; 7 Neurogen Inc., 1-1-52-201 Nakahozumi, Ibaraki 567-0034, Japan; 8 Center for Advanced Research and Education (CARE), Asahikawa Medical University, 2-1-1-1 Midorigaoka-Higashi, Asahikawa, Hokkaido, 078-8510, Japan; 9 DKTK Clinical Cooperation Unit Neurooncology, German Cancer Research Center (DKFZ), Im Neuenheimer Feld 280, Heidelberg, Germany; 10 Department of Neurology, University Medical Center Mannheim, Heidelberg University, Theodor-Kutzer-Ufer 1-3, Mannheim, Germany

## Abstract

The catabolism of tryptophan to immunosuppressive and neuroactive kynurenines is a key metabolic pathway regulating immune responses and neurotoxicity. The rate-limiting step is controlled by indoleamine-2,3-dioxygenase (IDO) and tryptophan-2,3-dioxygenase (TDO). IDO is expressed in antigen presenting cells during immune reactions, hepatic TDO regulates blood homeostasis of tryptophan and neuronal TDO influences neurogenesis. While the role of IDO has been described in multiple immunological settings, little is known about TDO’s effects on the immune system. TDO-deficiency is neuroprotective in C. elegans and Drosophila by increasing tryptophan and specific kynurenines. Here we have determined the role of TDO in autoimmunity and neurodegeneration in experimental autoimmune encephalomyelitis (EAE), a model of multiple sclerosis. We created reporter-TDO mice for *in vivo* imaging to show that hepatic but not CNS TDO expression is activated during EAE. TDO deficiency did not influence myelin-specific T cells, leukocyte infiltration into the CNS, demyelination and disease activity. TDO-deficiency protected from neuronal loss in the spinal cord but not in the optic nerves. While this protection did not translate to an improved overt clinical outcome, our data suggest that spatially distinct neuroprotection is conserved in mammals and support TDO as a potential target for treatment of diseases associated with neurodegeneration.

Kynurenines, the catabolites of the essential amino acid tryptophan (trp), can modulate adaptive immune responses as well as neuronal survival. Tryptophan metabolism is initiated by the two main key enzymes indoleamine-2,3-dioxygenase (IDO) and tryptophan-2,3-dioxygenase (TDO)^1^,^2^. This metabolic pathway is best studied in the context of immune regulation conferred by IDO. IDO is ubiquitously expressed at low levels and strongly induced in antigen-presenting cells (APC) and stromal cells by proinflammatory stimuli such as the T-helper cell-1 (Th1) cytokine interferon-γ (IFN-γ) or toll like receptor ligands like poly I:C and lipopolysaccharide^3^,^4^. IDO activity suppresses T cell responses by both, deprivation of trp and accumulation of kynurenines: The depletion of trp activates the *elongation initiation factor 2a* (EIF2a) kinase *general control non-derepressable 2* (GCN2), which initiates the amino acid starvation response in T cells, resulting in T cell suppression^5^. The secreted kynurenines, and especially the metabolite kynurenine (kyn), are capable of inhibiting T cell responses through activation of the *aryl hydrocarbon receptor* (AHR)^6^,^7^,^8^,^9^,^10^,^11^. IDO promotes tumor-associated immune suppression in animal models of cancer^11^,^12^,^13^. Consequently, pharmacological inhibitors of IDO are currently in clinical trials with the aim of enhancing antitumor immunity^14^. In the mouse model of multiple sclerosis (MS), experimental autoimmune encephalomyelitis (EAE), IDO fosters Th cell differentiation towards a regulatory phenotype and its inhibition or genetic ablation exacerbates the disease^15^,^16^,^17^.

Similar to IDO, TDO catabolizes trp although its expression pattern is fundamentally different. TDO is constitutively expressed in the liver, controlling energy homeostasis^18^. In addition it is expressed in several other cells and organs, including the placenta^19^, the pregnant uterus^20^, maternal and embryonic tissues in early conception^21^, epididymis, testis^22^ and neurons^23^. The expression pattern in different neuronal subsets and the function of neuronal TDO is a matter of intensive research^24^,^25^. TDO-deficient (TDO−/−) mice develop normally but display alterations in neurogenesis and anxiety-related behavior^26^. TDO-deficiency or inhibition is neuroprotective in Drosophila and C*. elegans*^27^,^28^,^29^. Glial tumors have been shown to express TDO, thereby promoting tumor cell survival and invasiveness and suppressing antitumor immune responses^10^,^11^.

There is limited data on the relevance of TDO-mediated trp catabolism on inflammation and neurodegeneration in mammals. We have used TDO-deficient mice and newly designed TDO reporter mice to study its role in the mouse model of MS, a disease characterized by chronic inflammation, demyelination and neurodegeneration.

## Results

### Cloning and characterization of the reporter-TDO mouse

**Figure 1:**
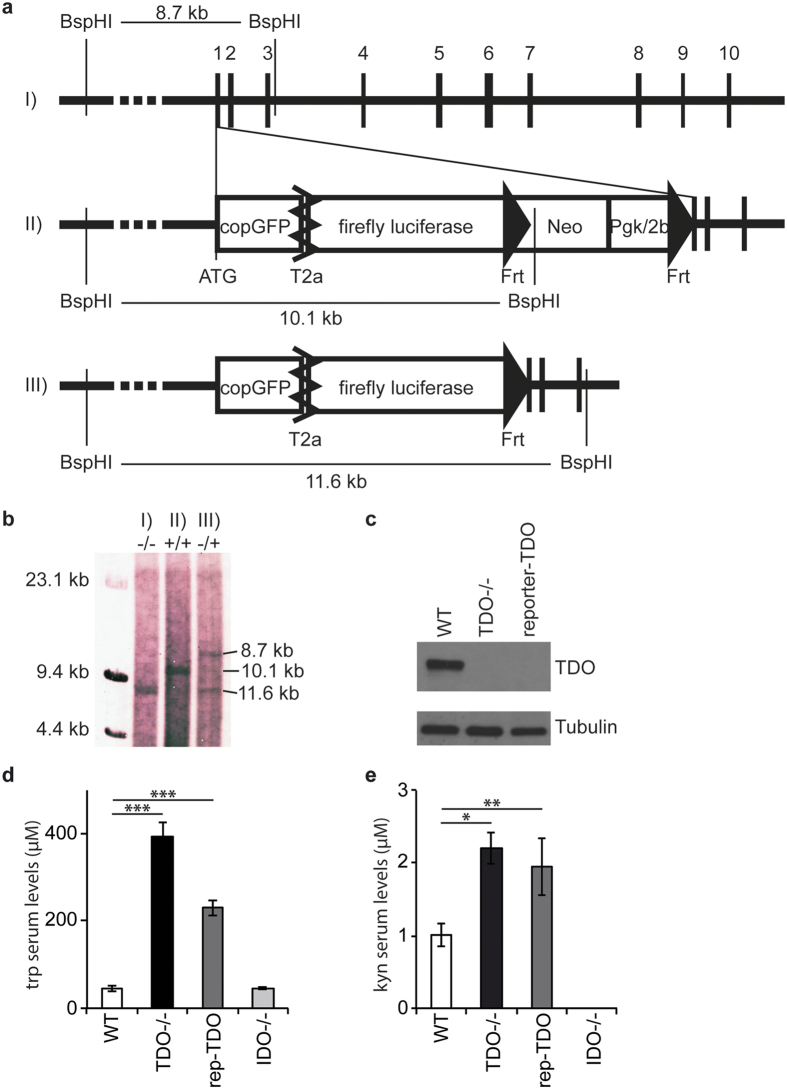
Cloning and phenotype of reporter-TDO mice. (**a**) Design of the transgenic construct for reporter-TDO mice including the southern blot strategy. (I) WT TDO2 gene. (II) Reporter cassette inserted into TDO2 locus. (III) Transgenic state after site-directed recombination by flippase. Digits 1–10: Exons of the TDO2 gene; BspHI: restriction sites for southern blot with annotated band sizes; ATG: start codon of TDO2 and reporter cassette; copGFP: copGFP gene; firefly luciferase: firefly luciferase gene; Neo: Neomycin cassette; Pgk/2b: Pgk/2b promoter; T2a: T2a site. Frt: flippase recognition target site. (**b**) Southern blot analysis of mouse DNA from transgenic mice with the genotypes explained in (**a**): (I) −/− (homozygous WT), (II) +/+ (homozygous transgenic) and (III) −/+ (hemizygous transgenic after Flp-Frt recombination). (**c**) Western blot analysis from hepatic lysate of WT, TDO−/− and homozygous reporter-TDO mice. One representative western blot of three independent experiments is shown (for uncropped gels see Supplementary Fig. 1). (**d**,**e**) HPLC measurements of serum levels of trp (**d**) and kyn (**e**) in naive WT, TDO−/−, reporter-TDO and IDO−/− mice. Means ± standard deviations of 3 mice per group are shown. *p < 0.05, **p < 0.005, ***p < 0.0001 according to unpaired Student’s t-test.

To determine the spatial and temporal regulation of TDO we generated reporter mice for TDO expression (reporter-TDO). We inserted a copGFP and a firefly luciferase gene into the TDO2 locus, starting at the start codon of the TDO2 gene (Fig. 1a). The construct was electroporated into embryonic stem cells of C57BL/6N mice. Accurate insertion of the transgene was assured by southern blot analysis (Fig. 1b). A stop codon at the end of the reporter construct prevents TDO2 transcription. Western blot analysis shows that homozygous reporter-TDO mice are deficient in hepatic TDO (Fig. 1c, Supplementary Fig. S1). Hemizygous and homozygous reporter-TDO mice do not show any phenotype in organogenesis and breeding behavior. HPLC measurements revealed that serum trp and kyn levels are comparable to previously described TDO-deficient mice (TDO−/−)^26^: Trp levels are elevated 8.8-fold in TDO−/− mice and 5.1-fold in homozygous reporter-TDO mice (Fig. 1d), kyn levels 2.2- and 1.9-fold, respectively (Fig. 1e). Elevated kyn levels in TDO−/− mice have been described before and it has been hypothesized that peripheral IDO accounts for this elevation^26^. Indeed, qPCR analysis shows that IDO expression is elevated in spleens but not in lymph nodes of TDO−/− mice (Supplementary Fig. S2a,b). Hepatic IDO expression is not altered in TDO−/− mice and expression levels are by two orders of magnitude lower than in lymphoid organs (Supplementary Fig. S2c). The above hypothesis is further substantiated by our measurements of trp and kyn in IDO-deficient mice (IDO−/−) (Fig. 1d,e): while serum trp levels are similar in WT and IDO−/− mice, kyn levels are below detection limits of our HPLC set-up (0.5 uM), indicating that hepatic TDO maintains systemic trp levels and hepatic kyn is rapidly further metabolized, while systemic kyn levels are produced by IDO.

### Hepatic TDO is induced during inflammation

**Figure 2:**
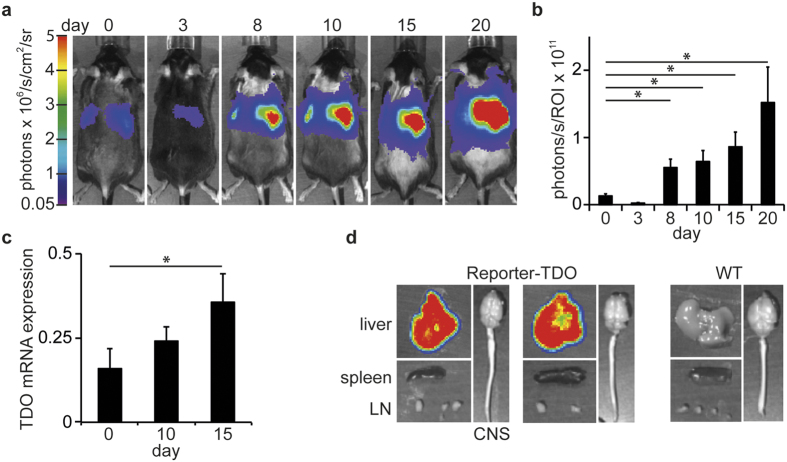
TDO regulation during EAE. (**a**) Luciferase imaging of one representative reporter-TDO mouse at indicated time points (days) after immunization. (**b**) Statistical evaluation of luciferase imaging, 5 mice per group. Means ± SEM are shown. *p < 0.05 according to Student’s t-test. (**c**) TDO mRNA expression relative to GAPDH as measured by qPCR analysis in hepatic tissue at indicated time points (days) after immunization. Means ± SEM are shown from 3 mice per time point. *p < 0.05 according to Student’s t-test. (**d**) Luciferase imaging of single organs of 2 representative of 5 reporter-TDO mice (left) and one WT mouse (right), injected on day 21 with luciferin and imaged 25 minutes thereafter.

We detected hepatic luciferase activity in reporter-TDO mice, which increased during the course of EAE (Fig. 2a,b). This was confirmed on the transcriptional level by qPCR analysis from liver tissue of immunized WT mice at the indicated time points (Fig. 2c). The effect is not MOG35–55 specific but a response to the induced inflammation, as reporter-TDO mice immunized with Complete Freund’s Adjuvant (CFA) alone emit similar levels of bioluminescence (Supplementary Fig. S3a,b). It is also not a consequence of weight loss and the integrated stress response (ISR) as starvation for three days, a well-known inducer of the ISR, did not enhance but rather suppress TDO expression (Supplementary Fig. S3c,d). No luciferase could be detected in the CNS, spleens and lymph nodes of immunized mice (Fig. 2d). Of note, qPCR analysis of lymph nodes, spleens, brains, cerebella and spinal cords revealed that TDO2 is expressed in lymphoid and neuronal tissue, although at levels three to four orders of magnitude lower than in the liver (Supplementary Fig. S4). No GFP expression was detected in the liver or CNS of the same mice, possibly due to posttranslational silencing of copGFP in the reporter construct.

### The antigen-specific T cell response is unaltered in TDO-deficient mice

**Figure 3:**
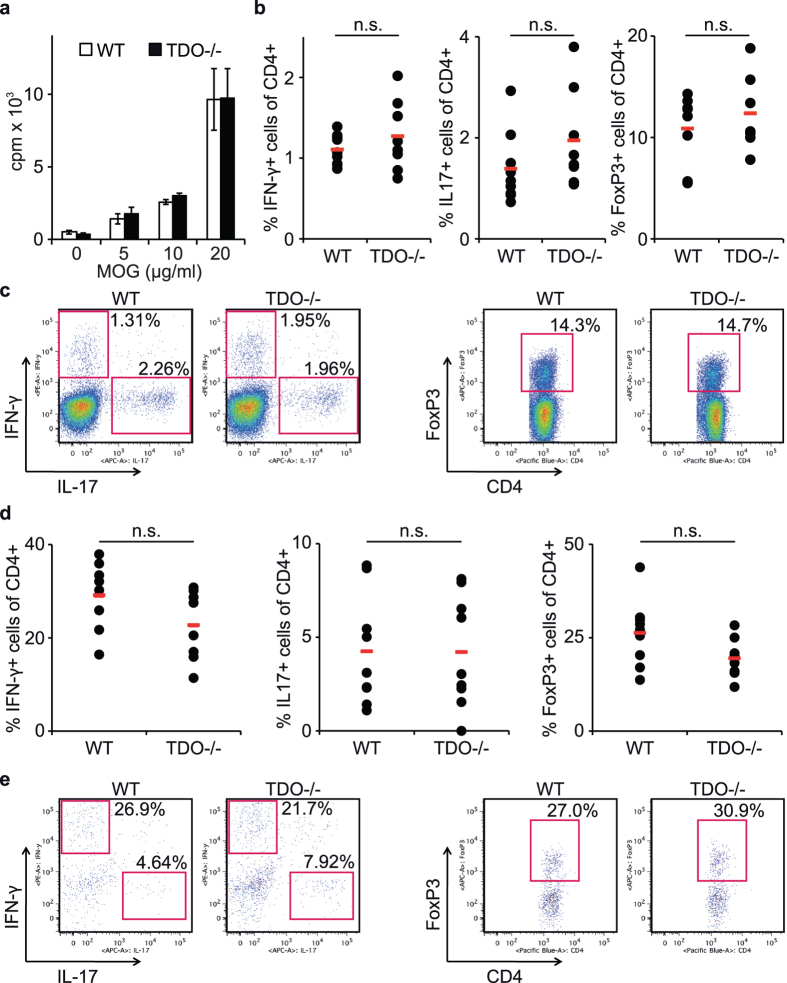
TDO deficiency does not influence T cell activation and differentiation. (**a**) Antigen-specific proliferation of T cells from WT vs. TDO−/− mice. T cells were isolated from lymph nodes 9 days after immunization and restimulated with MOG peptide 35–55. Proliferation was measured by ^3^H-thymidine intake. Representative results are shown of one experiment out of three independent experiments with three mice per group, respectively. Each measurement was carried out in triplicates. Means ± SEM are shown. No statistically significant differences between groups according to unpaired Student’s t-test. (**b**–**e**) Intracellular flow cytometry analyses of IFN-γ, IL-17 and FoxP3 in CD4+ T cells from (**b**,**c**) lymph node cells and (**d**,**e**) CNS tissue of immunized WT and TDO−/− mice. Each data point represents one individual measurement from three independent experiments. (**c**,**e**) Representative dot plots for (**b**,**d**). n.s.: not significant according to unpaired Student’s t-test.

When comparing *in vitro* T cell proliferation from immunized WT and TDO−/− mice, no significant differences were detected (Fig. 3a). ELISA measurements revealed that cytokine profiles of T cells isolated from draining lymph nodes of immunized mice were not altered by TDO deficiency (Supplementary Fig. S5). These findings were substantiated by intracellular flow cytometry of T cells from lymph nodes of WT and TDO−/− mice (Fig. 3b,c) and CNS infiltrating CD4+ T cells (Fig. 3d,e), which did not show any shift in Th cell differentiation. This indicates that TDO deficiency does not impact the pathogenic T cell response during neuroinflammation.

### TDO does not affect immune cell infiltration and glial activation in the CNS

**Figure 4:**
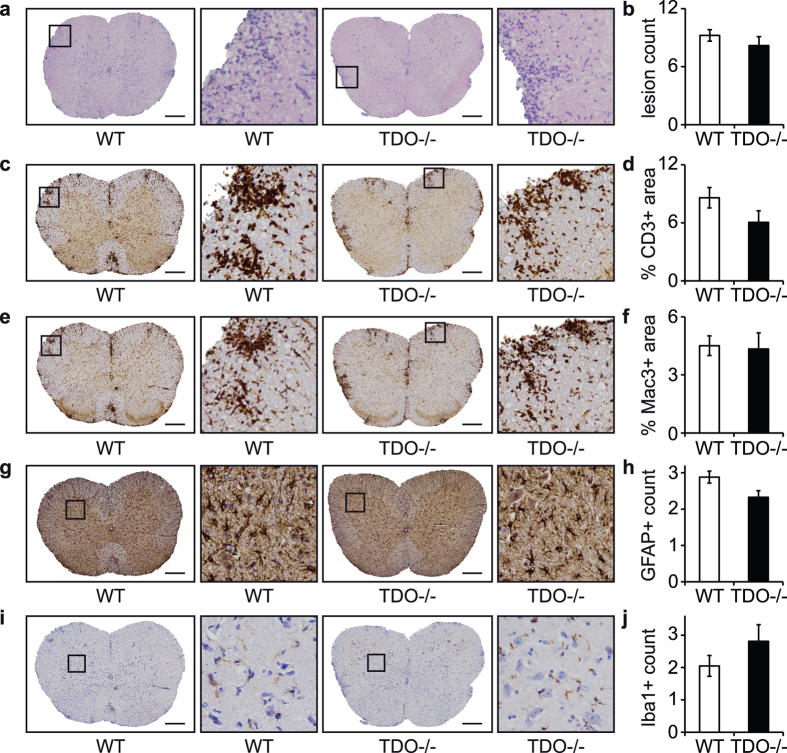
TDO deficiency does not change CNS infiltration and glial activation. Immunohistochemistry images of spinal cord slides from WT and TDO−/− EAE mice sacrificed at the peak of disease. (**a**) representative HE stainings, (**b**) infiltrative lesion load per slide, (**c**) representative CD3 stainings, (**d**) % CD3+ stained area per slide, (**e**) representative Mac3 stainings, (**f**) % Mac3+ stained area per slide, (**g**) representative GFAP stainings, (**h**) GFAP+ cell count per defined area of the slide, (**i**) representatitve Iba1 stainings, (**j**) Iba1+ cell count per defined area of the slide. Mice were sacrificed on day 14 after immunization. At least 3 spinal cord slices of each mouse were analyzed with 6 mice per group. Statistical analysis was achieved by blinded investigators: (**b**) manual count of infiltrative lesions per slide, (**h**,**j**) manual count of single cells per designated area, (**d**,**f**) computational analysis of stained area per slide using FIJI/ImageJ software. Means ± SEM are shown. No statistically significant differences could be detected according to unpaired Student’s t-tests. Scale bars: 200 μm.

We next assessed whether TDO influences immune cell infiltration and glial activation in the CNS, both hallmarks of MS- and EAE pathology. Immunohistochemistry revealed no significant difference between WT and TDO−/− animals with respect to inflammatory lesion load (Fig. 4a,b), infiltrating CD3+T cells (Fig. 4c,d) or Mac3+ myeloid cells (Fig. 4e,f). Also glial activation remained unchanged, as shown by immunohistochemistry for the astroglial marker GFAP (Fig. 4g,h) and the microglial marker Iba1 (Fig. 4i,j).

### TDO does not change demyelination and clinical disease activity in EAE

**Figure 5:**
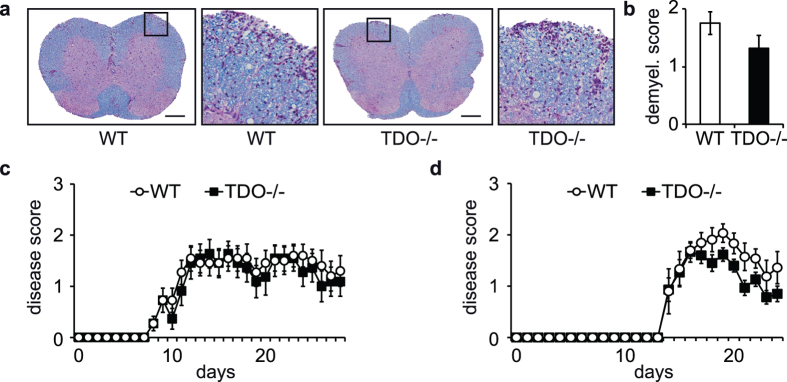
TDO deficiency does not influence demyelination and clinical scores. (**a**) Representative Luxol-PAS stainings of spinal cord slides from WT and TDO−/− EAE mice sacrificed at the peak of disease. (**b**) Demyelinated area (counts) per slide. Mice were sacrificed on day 14 after immunization. At least 3 spinal cord slices of each mouse were analyzed, 6 mice per group. Statistical analysis was achieved by manual count of demyelinated lesions according to a demyelination score, Scale bars: 200 μm. (**c**,**d**) Clinical disease scores are shown from EAE mice (**c**) WT vs. TDO−/− with normal immunization protocol (MOG 200 μg/mouse), (**d**) WT vs. TDO−/− with low-dose immunization protocol (MOG 50 μg/mouse). Each panel shows one representative experiment out of at least three independent experiments. No statistically significant differences could be detected according to Mann-Whitney-U test.

In line with these data, myelin degradation remained unchanged in TDO-deficient mice, as determined by Luxol-PAS staining (Fig. 5a,b). Accordingly, there were no differences in clinical disease activity between WT and TDO−/− mice as measured by the degree of paralysis (Fig. 5c). Even when using a weaker immunization protocol resulting in a later onset and a milder disease course, the clinical disease activity was not aggravated by TDO-deficiency (Fig. 5d).

### Neuroprotective vs. neurotoxic effect of TDO

**Figure 6:**
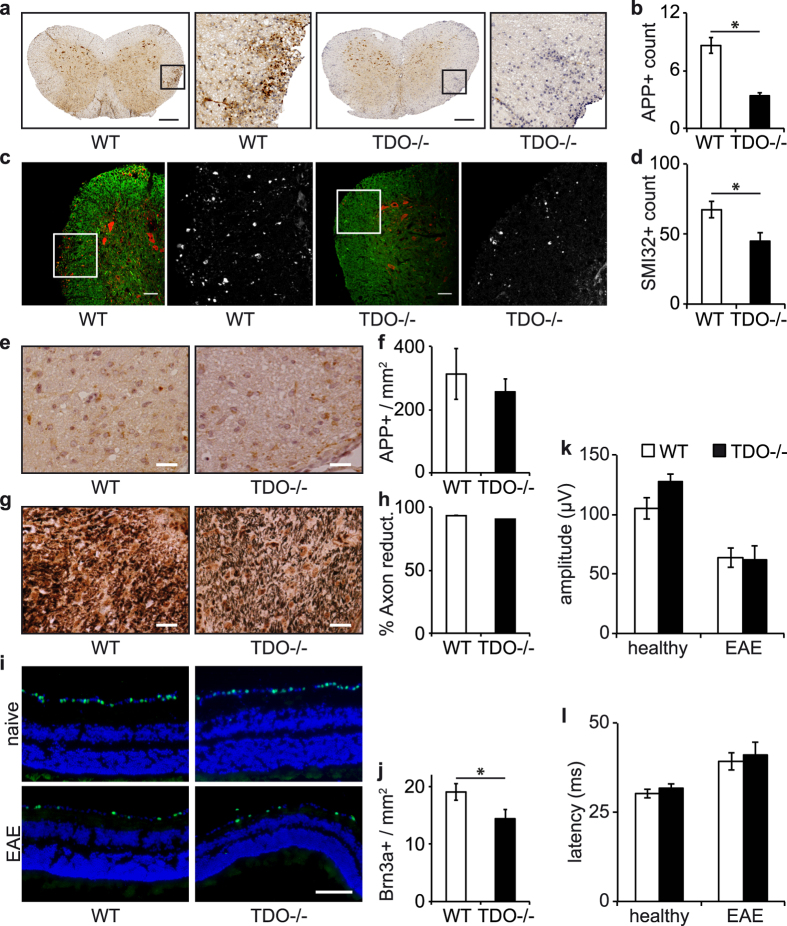
TDO deficiency alters neuronal survival. (**a**,**c**) Representative immunohistochemistry images of spinal cord slides from WT and TDO−/− EAE mice sacrificed on the peak of disease. (**a**) Representative APP immunohistochemistry images, (**b**) APP+ counts per slide. (**c**) Representative SMI-32 immunohistochemistry images, red: SMI-32, green: MBP, white: magnified areas showing the raw SMI-32 images, (**d**) SMI-32+ counts per slide. At least 3 spinal cord slices of each mouse were analyzed, 6 mice per group. APP+ and SMI-32+ cells per designated area were counted manually by blinded investigators. *p < 0.05 according to unpaired Student’s t-test. Scale bars: 200 μm. (**e**,**g**) Representative Immunohistochemistry images of optic nerves and (**i**) retinas from WT and TDO−/− EAE mice sacrificed on day 21 of EAE. (**e**) Representative APP immunohistochemistry, (**f**) APP+ counts per mm^2^, (**g**) representative Bielschowsky stainings, (**h**) % Axon reduction per slide. (**i**) Representative Brn3a stainings of retinas, green: Brn3a+ RGC, blue: DAPI, (**j**) Brn3a+ counts per mm^2^. Statistical analysis was performed from at least 10 sections per optic nerve/retina from one representative of 2 independent experiments with 8 mice per group, *p < 0.05 according to unpaired Student’s t-test, Scale bars: 25 μm. (**k**) Amplitudes and (**l**) latencies of VEP measurements in WT (white) and TDO−/− (black) EAE mice. Measurements were performed before immunization and on day 21 of EAE. Mean ± SEM are shown of 10 mice per group.

As some kynurenines elicit excitotoxic and others neuroprotective effects, we analyzed signs of axonal degeneration. Immunohistochemistry of inflamed spinal cords revealed reduced numbers of β-amyloid precursor protein (APP) positive axons in TDO−/− mice (Fig. 6a,b), which accumulates in the terminal axons during Wallerian degeneration. This indicates increased neuronal survival in TDO−/− mice, despite similar levels of inflammation and demyelination as well as degree of paralysis. We confirmed this subtle neuroprotective effect with immunohistochemistry of spinal cord slices for non-phosphorylated neurofilament (SMI-32), a stress marker for myelinated axons^30^ (Fig. 6c,d).

To analyze the spatial specificity of this effect, we looked at the optic nerves, a second distinct neuronal compartment frequently affected in MS. Here, immunohistochemistry for APP and Bielschowsky’s silver impregnation did not show any differences between TDO-deficient and WT mice (Fig. 6e–h). Interestingly, staining of the retinal ganglion cell marker (RGC) Brn3a revealed reduced numbers of RCGs in TDO−/− EAE mice (Fig. 6i,j). Functional testing of the optic tract using visual evoked potentials (VEPs), however, did not reveal relevant differences (Fig. 6k,l). Collectively, these data indicate that TDO controls spatially distinct neurodegeneration in autoimmune neuroinflammation, albeit at a subclinical level.

## Discussion

IDO-mediated trp metabolism suppresses antigen-specific T cell immunity and innate immunity in models of allergy, asthma, infection, cancer and autoimmunity^31^,^32^,^33^ including EAE^15^,^16^,^17^, where natural and synthetic kynurenines modulate antigen-specific T cell responses^34^. Recently, we and others have shown that TDO-driven tryptophan catabolism in glioblastomas is equally capable of suppressing antitumor immune responses^10^,^11^. In addition, several kynurenines are well-described neuroactive metabolites. As TDO expression has been observed in distinct neuronal compartments it was tempting to hypothesize that TDO might impact immune responses and neuronal survival in a model of autoimmune neuroinflammation.

Our study shows increased hepatic expression and activity of TDO during EAE. However, our data do not support a role of TDO in modulating antigen-specific T cell responses in the EAE model. The discrepancy between IDO and TDO can be explained by different mechanisms. Most obvious are the divergent expression profiles of the two enzymes: *In vivo* bioluminescence analyses of reporter-TDO mice indicate that TDO is mainly expressed in the liver while the signal strength was not sufficient to detect TDO expression in lymphoid and neuronal tissue, which is by three to four orders of magnitude lower than the hepatic expression levels. It has been shown that within the CNS TDO expression is restricted to distinct cell subsets such as hippocampal neurons^24^,^25^ (http://mouse.brain-map.org/experiment/show/70565321). In contrast, IDO is expressed ubiquitously and strongly induced in APCs by proinflammatory mediators and it changes trp and kyn levels in the local micromileu. IDO is highly expressed in the induction phase of EAE not only in secondary lymphatic organs but also in the spinal cord and several areas of the brain^16^,^17^. The divergent roles of IDO and TDO are supported by our HPLC measurements of trp and kyn serum levels in TDO−/− and IDO−/− mice: hepatic TDO is the main trp metabolizing enzyme which maintains systemic trp levels. Hepatic kyn is very likely rapidly metabolized. IDO in turn is responsible for producing kyn in the peripheral tissue and thereby for serum kyn levels. Its mRNA expression is elevated in spleens of TDO−/− mice. IDO tightly controls kynurenine levels in the local microenvironment, but it does not change systemic trp levels. This is supported by previous studies which found that IDO inhibition and IDO deficiency exacerbated EAE regardless of kyn serum levels^15^.

IDO accumulates kyn but depletes trp in the extracellular compartment, which sustains two synergistic mechanisms: Trp depletion leads to immunosuppression via the initiation of the amino acid starvation response by GCN2, which senses amino acid deficiency through uncharged tRNA. Its effects on immune reactions is best studied in CD4+ and CD8+ T cells where trp depletion creates T cell anergy^5^. Accordingly, chronic neuroinflammation is exacerbated in GCN2-deficient mice^35^,^36^. At the same time accumulation of kyn and downstream metabolites in the local microenvironment leads to immunosuppression by activating the AHR, an immunoregulatory receptor which is best studied on T cells in EAE and tumor models^6^,^7^,^8^,^10^,^37^. In contrast, hepatic TDO deficiency raises both, serum levels of trp and kyn in the serum, possibly resulting in two opposing immunoregulatory mechanisms.

Kynurenic acid is a well known neuroprotective factor while other kynurenines like 3-hydroxykynurenine and quinolinic acid are excitotoxins^29^,^38^,^39^,^40^,^41^. It has been shown that TDO deficiency elevates merely all kynurenines in serum and brain tissue as demonstrated for kyn, kynurenic acid, 5-hydroxyindoleacetic acid, indolelactic acid and indoleacetic acid^26^. TDO-deficient mice display a mild phenotype with subtle alterations in anxiety-related behavior^26^. Recent studies in flies and lower vertebrates confirmed the neuroactive role of TDO: TDO inhibition and accumulation of kynurenic acid is neuroprotective in a Drosophila model of Huntington’s and Alzheimer’s disease^27^,^29^ and in *C. elegans* TDO-deficiency extends the animal’s lifespan by suppressing proteotoxicity^28^. Here, it also appears that the systemic rather than the local CNS dysbalance of trp metabolites is responsible for the phenotype.

Our results extend these observations to the mammalian system and support the concept that increasing systemic trp and its metabolites result in a net neuroprotective phenotype. In addition to our observation that this phenotype in the EAE model is subtle and at a subclinical level, we found distinct effects on neurons of different systems: While there is a protective effect of TDO deficiency in spinal axons, this finding was not recapitulated in neurons of the visual system, rather the contrary, we saw a slight but significant additional neurotoxic effect on Brn3a+ RGC in TDO−/− mice.

This divergent effect could be explained by i) differential susceptibility to neurotoxicity and ii) differential expression of TDO in certain areas of the brain. Differential susceptibility to excitotoxicity induced by the trp metabolites quinolinic acid and 3-hydroxykynurenine is mainly determined by distinct expression patterns of NMDA receptor subunits^42^. Indeed, RGC express functional NMDA receptors^43^, and while early reports described their relative resistance to excitotoxins^44^, more recent research showed that this resistance applies mainly to the subset of OPN4+ intrinsically photosensitive RGCs, whereas Brn3a+ traditional RGCs are susceptible to excitotoxicity by NMDA^45^. Spinal neurons, while expressing NMDA receptors and being responsive to glutamate, show only low excitability to quinolinic acid^46^, and therefore the protective effects of kynurenine and kynurenic acid may prevail. In contrast, hippocampal and striatal neurons are excitable by quinolinic acid^46^, (while at the same time expressing TDO, see below). In addition to excitotoxicity via direct NMDA receptor activation, quinolinic acid can be toxic to neurons and glial cells (i) by increasing microenvironment glutamate concentration via inhibiting glutamate release by neurons and its uptake by astrocytes, (ii) by potentiating the effect and toxicity of other NMDA receptor antagonists, and iii) by formation of reactive oxygen species which cause lipid peroxidation (for a comprehensive review, see ref. 47).

TDO expression varies highly between different neurons with by far the highest expression in granule cells of the hippocampus^24^,^25^ (http://mouse.brain-map.org/experiment/show/70565321). Inflammatory lesions, demyelination and neurodegeneration in our EAE model are mainly restricted to the spinal cord and the cerebellum^48^. In human MS however, a majority of inflammatory lesions are located in paraventricular white matter, marked hippocampal atrophy is characteristic to the disease^49^ and cognitive impairment is present already at early stages of MS^50^. Therefore, TDO inhibition could prove to be more relevant in human MS than what we might deduce from its mouse model.

In summary, our study suggests that suppressing systemic TDO activity – as previously suggested in worms and flies – may represent a viable neuroprotective strategy in diseases associated with neurodegeneration such as multiple sclerosis.

## Methods

### Animals

Transgenic TDO-deficient mice (B6.Tdo2tm1Tnak, here referred to as “TDO−/−”)^26^ on a C57BL/6N background were provided by Hiroshi Funakoshi, IDO-deficient mice (B6.129-Ido1tm1Alm/J, here referred to as “IDO−/−”)^51^ on a C57BL/6J background were purchased from Jackson Laboratories. C57BL/6N mice were obtained from Charles River Laboratories (Sulzfeld, Germany) and used as age- and sex-matched WT control animals. Transgenic reporter mice for TDO (B6-Tdo2tm#(GFP/Luc)/Dkfz, here referred to as “reporter-TDO”) were created on a C57BL/6N background with JM8A1.N3 embryonic stem (ES) cells. All used animals were female and 8–12 weeks old. Animals were housed in the SPF animal facility of the German Cancer Research Center (DKFZ) in Heidelberg, Germany. All animal work was performed in accordance with the European and German animal protection law with approval from the “Regierungspräsidium” in Karlsruhe, Germany and the “Tierlaborausschuss” of the German Cancer Research Center (DKFZ) in Heidelberg, Germany.

### Cloning of the transgenic reporter-TDO mouse

The transgenic construct containing a copGFP and a firefly luciferase gene in the TDO2 locus, starting at its start codon (Fig. 1a), was cloned using an artificial chromosome (BAC) and Red/ET technology^52^,^53^. A stop codon was inserted at the 5′ end of the insert, therefore a homozygous mouse results in a knockout of TDO. Red/ET cloning was carried out by GeneBridges, Heidelberg, Germany. The BAC RP23–36B19 was subjected to PCR analysis for identity and integrity and cloned into a high-copy plasmid backbone, then it was again checked by restriction digest. In a first step copGFP/firefly luciferase was inserted into the plasmid by multi-site Red/ET recombination. In a second step, a kanamycin/neomycin resistance cassette was included again by multi-site Red/ET recombination. As only very few plasmid copies in a cell undergo Red/ET recombination very little DNA of a mosaic positive clone was used to transform new cells. A positive clone was identified by restriction digest and sequenced. The plasmid was linearized using the restriction enzymes SalI and SacII before electroporation into ES cells.

### Embryonic stem cell culture

2 × 10^6^ JM8A1.N3 ES cells were cultured on inactivated feeder cells plated on the day before. After 5 days of culture cells were trypsinized and electroporated with 50 μg of the DNA construct using a gene pulser electroporation system (BioRad, Hercules, CA, USA). After another 24 h of cell culture ES cells selection started by addition of gentamycin (G-418, Sigma-Aldrich, Seelze, Germany). After 7 days of culture under selection conditions, appr. 350 clones were picked and further cultured in 48 well plates for two more days under selection. Then cells were trypsinized and 2/3 were frozen for later blastocyst injection while 1/3 was cultured on gelatinized 24-well plates for another 6–10 days for southern blot analysis.

### Blastocyst injection

Superovulation of 4–8 week old female mice was achieved by i.p. injection of 7 IU pregnant mare’s serum gonadotropin and 46–48 h later by 7 IU human chorionic gonadotropin. 1 h after the last injection mice were fertilized by congenic males and checked for a vaginal plug. Blastocysts were obtained at day 3.5 by flushing of the isolated oviduct/uterus. Transgenically altered embryonic stem cells were injected into the blastocoel. Blastocysts were injected into the uterus of pseudopregnant foster mothers (after mating with vasectomized males). Genotyping of the first transgenic/mosaic litter was achieved by fur color. Positive mice were bread with WT C57BL/6N mice and germline transmission was screened by PCR and southern blotting. Transgenic mice were crossed to Flp-deleter mice (B6.CG-TG(ACTFLPe)9205Dym) to erase the neomycin cassette. Correct insertion of the reporter cassette was checked by southern blotting before and after Flp deletion (Fig. 1b). Homozygous females were used for bioluminescence imaging.

### Southern blot analysis and PCR genotyping

Southern blot strategy including the expected band sizes is depicted in Fig. 1a. Southern blot analysis was achieved according to standard protocols using a DIG DNA Labeling and Detection Kit (Roche, Basel, Switzerland). Briefly, a 1049 bp template DNA was amplified using the primers: forward: 5′-GGAAGGGGTGGAAACCAGAGGC-3′ and reverse: 5′-AGAGGAAGTTGTTCGGACGCTGC-3′. The template was labeled with Digoxigenin-11-dUTP. Genomic mouse DNA was isolated from embryonic stem cells or tails by incubation at 56 °C for 2 h with 1 μg/ml Proteinase K (Sigma) in DNA lysis buffer, containing 100 mM Tris-HCl, 200 mM NaCl, 5 mM EDTA and 0.2% SDS, followed by an inactivation step at 95 °C for 20 min. Genomic DNA was precipitated with isopropanol and washed with alcohol, then 10 μg were digested using BspH1 (New England Biolabs, Ipswich, MA, USA) in NEB Buffer 4 overnight before electrophoresis on a 1% agarose gel. DNA was denaturated on the gel by incubation in 0.5 M NaOH/1 M NaCl for 0.5 h, rinsed and neutralized twice with 0.5 M Tris, pH7. 4/3 M NaCl for 20 min. The gel was placed in 20× saline-sodium citrate buffer with a nitrocellulose membrane on top. DNA transfer onto the membrane was achieved by capillary transfer overnight. The membrane was dryed at 80 °C for 2 h and hybridized with the DIG-labeled template in 4 × SSPE, 5× Denhardt’s reagent, 1% SDS, 100 μg/ml salmon sperm DNA and 1% blocking reagent at 65 °C. The membrane was washed 3×, blocked for 0.5 h with blocking reagent, incubated with anti-DIG solution (1:5000), then washed and incubated for several hours in NBT and Bcip until DNA staining became visible.

After confirmation by southern blot analysis subsequent mouse generations were genotyped by standard PCR using the following primers: fwd: 5′-AGCTGGGTGCTGATTGGCTGTG-3′, rev1: 5′-CGCCGTCCTCGTACTTCTCGAT-3′, rev2: 5′-AGGCTAAGGCTGGAAAGTTCCCT-3′ (expected amplicons: transgen: 408 bp, WT: 234 bp). Flippase efficiency was confirmed by PCR using the following primers: fwd: 5′-CGGCCGCTTCGAGCAGACAT-3′, rev: 5′-AGGCTAAGGCTGGAAAGTTCCCT-3′ (expected amplicon after Flp recombination: 404 bp).

### Monitoring of phenotype

The following parameters have been monitored in homozygous reporter-TDO mice in particular: breeding behavior, litter size, litter per year, surviving litter size as well as weight and size after birth until week 4. Macroscopic autopsy has been performed on 5 adult mice at the age of 10–12 weeks with a special focus on abdominal organs (liver, spleen, stomach, intestine, kidneys, pancreas, bladder) as well as lung, heart and brain, and they showed no abnormalities in appearence, shape and weight. As TDO is mainly expressed in the liver, we examined hepatic H&E stainings, also showing no difference to livers from WT mice.

### Western blot analysis

Whole-liver lysates were prepared with high-speed ball mill (Tissue Lyser II, Qiagen, Venlo, Netherlands) in ice cold tris (hydroxymethyl) aminomethane hydrochloride (TRIS-HCl, 50 mM, pH 8,0; Carl Roth GmbH, Karlsruhe, Germany) containing 150 mM NaCl (J.T. Baker, Deventer, Netherlands), 1% Triton X-100 (Appli-Chem GmbH, Darmstadt, Germany), 10 mM EDTA (Gerbu Biotechnik, Gaiberg, Germany), 200 mM dithiothreitol (Carl Roth) 3% 2-mercaptoethanol (Sigma), 100 μM phenylmethylsulfonyl fluoride, 10 μg/mL aprotinin and 5 μg/mL leupeptin (Carl Roth) and centrifuged at 4 °C (10 min, 5000 g). The protein concentration of the supernatants was determined using the Bio-Rad protein assay (Bio-Rad, Hercules, CA, USA) at 595 nm. The desired amount of protein (30 μg per lane) was separated by 10% sodium dodecyl sulfate–polyacrylamide gel electrophoresis and transferred to a 0.2 μm-pore nitrocellulose membrane (Whatman, Dassel, Germany). After 1 h of blocking in phosphate-buffered saline supplemented with 0.1% Tween 20 (Sigma) and 5% powdered milk (Carl Roth), the membrane was incubated with rabbit anti-TDO2 antibody (1:750; Novus Biologicals, Minneapolis, MN, USA) or goat anti-GAPDH (1:2000; Abcam, Cambridge, UK) or mouse anti-alpha-tubulin (1:2000; Sigma) as loading control overnight at 4 °C. After a 1 h incubation at 21 °C with the secondary antibodies HRP-conjugated donkey anti-rabbit (1:5000; GE Healthcare, Buckinghamshire, UK), HRP-conjugated donkey anti-goat (Santa Cruz Biotechnology Inc., Santa Cruz, CA, USA) or HRP-conjugated sheep anti-mouse (1:5000; GE Healthcare, Buckinghamshire, UK), protein detection was performed using enhanced chemiluminescence Plus reagent (GE Healthcare).

### High Performance Liquid Chromatography (HPLC) measurement of kyn and trp in serum samples

For protein precipitation each 300 μl serum sample was treated with 50.6 μl of 72% trichloroacetic acid. Samples were centrifuged at maximum speed for 12 min and the supernatants were frozen overnight at −20 degrees. After thawing the samples, the centrifugation step was repeated and the obtained supernatants were used for HPLC analysis. A Dionex Ultimate^®^ 3000 uHPLC (Thermo Scientific, Waltham, MA, USA) was used for chromatographic separation of kyn and trp. This was achieved on a reversed phase Accucore™ aQ column (Thermo Scientific™) with 2.6 μm particle size with a gradient mobile phase consisting of 0.1% trifluoroacetic acid (TFA) in water (A) and 0.1% TFA in acetonitrile (B). Kyn and trp were detected based on comparison with standards, their retention times and UV emission spectra at 365 nm and 280 nm, respectively. Results were analyzed using the Chromeleon™ 7.2 Chromatography Data System (Thermo Scientific™ Dionex™).

### qRT-PCR

RNA was isolated using the Qiagen RNAeasy Plus RNA isolation kit (Qiagen, Venlo, the Netherlands) according to manufacturer’s instructions. cDNA was synthesized with the High Capacity cDNA Reverse Transcription Kit (Applied Biosystems, Waltham, MA, USA) using random hexamers. qRT-PCR for IDO was performed in 2 duplicated serial dilutions, on an ABI 7000 thermal cycler with SYBR Green PCR Mastermix (Eurogentec, Cologne, Germany). Primer sequences: IDO: fwd: 5′-GCTTTGCTCTACCACATCCAC-3′, rev: 5′-CAGGCGCTGTAACCTGTGT-3′, GAPDH: fwd: 5′-GCCTTCCGTGTTCCTACCC-3′. rev: 5′-CAGTGGGCCCTCAGATGC-3′. qRT-PCR for TDO was performed using TaqMan Gene Expression probes and TaqMan Fast Universal PCR Master Mix (ThermoFisher Scientific, Waltham, MA, USA), TDO2: Mm00451269_m1, GAPDH: Mm99999915_g1, detector: FAM. Data were evaluated with AB 7000 System SDS software. All results were normalized to GAPDH.

### Immunization of Mice

Mice were immunized s.c. with complete Freund’s Adjuvant (Difco/BD, Heidelberg, Germany) containing 200 μg of Mycobacterium tuberculosis (Difco/BD) in emulsion with myelin oligodendrocyte glycoprotein peptide 35–55 (MOG35–55) (Peptide Synthesis Core Facility of the DKFZ) at concentrations of 200 μg/mouse (normal EAE protocol), 50 μg/mouse (low dose EAE protocol) or without MOG35–55 (control experiment in Supplementary Fig. S3a,b). Mice received 200 ng/mouse pertussis toxin (List Biological Laboratories Inc., Campbell, CA, USA) i.p. on the day of immunization and 2 days thereafter. Clinical signs of disease were scored daily according to a standard scoring system (0, no clinical signs; 1, loss of tail tone; 2, hind limb weakness; 3, complete hind limb paralysis; 4, hind limb and forelimb paralysis; and 5, moribund or dead).

### Bioluminescence imaging and measurement of reporter-TDO mice

Hemizygous Reporter-TDO mice were assayed for bioluminescence intensity using an *in vivo* Imaging System (IVIS, Caliper/PerkinElmer, Waltham, MA, USA) that uses a cooled charged-coupled device (CCD) camera. 50 mg/kg d-luciferin (StayBrite™, BioVision, Mountain View, CA, USA) were injected 5 minutes before imaging, during which mice were anaesthetized with isoflurane. The imaging signal was quantitated as photons/s/cm2/steradian and integrated over 10 minutes, using Living Image software (version 2.50, Caliper/PerkinElmer). For images of single organs, mice were injected 5 minutes before sacrifice of the mice. Organs were prepared quickly and images could be taken after additional 25 minutes. For signal quantification, photons were obtained from equally sized and positioned regions. Twenty-four hours before immunization, baseline imaging was performed to ensure longitudinal comparison of bioluminescence. Bioluminescence was depicted as fold induction over baseline levels.

### Intracellular detection of cytokines by flow cytometry

For flow cytometric intracellular detection of cytokines lymph nodes were harvested 9 d after immunization and mechanically dissociated through a 70 μm cell strainer (BD Falcon), then washed with PBS and centrifuged at 1,200 rpm and 4 °C for 10 min. 10^6^ cells per well were seeded for 5 h in 24 well plates (TPP, Trasadingen, Switzerland) in enriched RPMI (Gipco/LifeTechnologies, Carlsbad, CA, USA) (containing 10% FBS, 100 U/ml penicillin, 0.1 mg/ml streptomycin, 1 mM Sodium Pyruvate, (all from PAA, Pasching, Austria), 25 mM Hepes, 5 × 10^−5^ M beta-mercaptoethanol (both from Sigma), 0.1 mM non-essential amino acids (Lonza, Basel, Switzerland) and 2 mM L-glutamine (Gibco/LifeTechnologies)) with 20 ng/mL PMA, 1 μg/mL ionomycin, and 5 μg/mL brefeldin A (all from Sigma). Cells were stained for CD4 (BioLegend, San Diego, CA, USA) and then permeabilized with cytofix/cytoperm solutions (BD Biosciences, San Jose, CA, USA) and stained for IFN-γ, IL-17, and FoxP3 (ebioscience, San Diego, CA, USA) according to standard protocols.

### T cell Proliferation and Cytokine Measurement

For the measurement of antigen-specific proliferation of T cells, mice were sacrificed on day 9 after immunization with MOG p35–55. Cells were mechanically singularized as described above and processed as described earlier^4^. Briefly, lymph node cells were seeded in U-bottom 96-well plates at 5 × 10^5^ cells per well in enriched RPMI, antigen specific activation was achieved by addition of 5–20 μg/mL MOG p35–55. For measurement of T cell proliferation, cells were pulsed after 72 h with 3H-methylthymidine (Amersham-Pharmacia Biotech, Amersham, UK) at 1 μCi per well for the last 18 h. Cells were harvested using a Tritium Harvester (Tomtec, Unterschleissheim, Germany) and a β-plate reader (Wallac/PerkinElmer, Wellesley, MA, USA) with BetaWin software. For cytokine measurements, supernatants were taken after 72 h of culture and measured by ELISA for the cytokines IL-2, IL-4, IL-6, IL-17A, and IFN-γ (ebioscience) and after 96 h for the measurement of IL-10 (ebioscience) according to the manufacturer’s instructions.

### Tissue preparation and immunohistochemistry of spinal cords

Mice were anesthetized by i.p. injection of 100 mg/kg ketamin and 20 mg/kg xylazin and then perfused with 50 mL of PBS by heart puncture. Brains and spinal cords were taken out and fixed in 4% (vol/vol) paraformaldehyde for 24 h and then transferred into 30% (wt/vol) sucrose for further storage. For immunohistochemistry spinal cords were cut into at least five parts and embedded into paraffin blocks to generate multiple axial slices. Immunohistochemistry was performed according to standard protocols^54^ using the following antibodies: CD3 (Dako/Agilent Technologies, Glostrup, Denmark), Mac3 (BD Pharmingen), GFAP (Merck Millipore, Billerica, MA, USA), Iba1 (Wako Diagnostics, Richmond, VA, USA), β-APP (Merck Millipore), SMI-32 (Covance, Princeton, NJ, USA). Hematoxylin and Eosin (H&E) stainings as well as Luxol-fast-blue/Periodic Acid-Schiff (Luxol-PAS) stainings were performed according to standard protocols. Microscopy images were taken using an NiE inverted automated microscope (Nikon, Tokyo, Japan), a Keyence BZ-9000 automated microscope and a LSM 700 confocal microscope (Zeiss, Jena, Germany).

### Histopathological analyses of spinal cords

Statistical evaluation of infiltrating immune cells was obtained from H&E stainings by manually counting lesions of infiltrating cells per slide. Demyelination was evaluated by manually counting demyelinated areas in luxol-PAS–stained spinal-cord slides, correlating it to the following score: 0.5, single demyelinated spot; 1, several spots; 2, confluent sites of demyelination; 3, demyelination of one half of the spinal cord; and 4, demyelination of more than half of the spinal cord^55^. GFAP and Iba1 immunohistochemistry was manually evaluated by counting the number of single stained cells with a corresponding nucleus per spinal cord slide. Immunohistochemistry images for APP and non-phosphorylated neurofilament (SMI-32) were manually evaluated by counting the number of APP-positive axonal profiles per spinal cord slide. CD3 and Mac3 immunohistochemistry was evaluated using Fiji/ImageJ software^56^, correlating the stained area to the whole spinal cord area. Morphological analyses and manual counting were carried out by a blinded investigator. All statistical evaluations were carried out in multiple slides of multiple animals as indicated in the corresponding figure legend.

### Optic nerve histopathology

Healthy mice and those at day 21 of EAEwere anesthetized with 200 mg/kg ketamin and 40 mg/kg xylazine i.p. and transcardially perfused with 4% paraformaldehyde in PBS. Optic nerves were dissected, processed for paraffin-embedding and 0.5 μm transverse sections were cut. Bielschowsky’s silver impregnation was performed in order to depict axonal pathology, as previously described^55^. To further assess axonal degeneration, immunohistochemistry was performed using an antibody against APP as described previously^57^.

### Retinal histopathology

Following perfusion (see above), eyes were enucleated, post-fixed in 4% paraformaldehyde for 2 hours and cryoprotected in 30% sucrose overnight. Eyes were then embedded in mounting medium (Tissue-Tek O.C.T Compound, Sakura Finetek Europe, Alphen aan den Rijn, Netherlands) and frozen. Twelve μm coronal sections were sliced for immunofluorescence. Sections were washed in PBS and blocked in 10% sera in 0.1% PBS-Triton X. Sections were incubated with anti Brn3a (Santa Cruz Biotechnology Inc, Santa Cruz, CA, USA) and staining was visualized with the an Alexa 555-conjugated secondary antibody (Jackson Immuno Research Laboratories, West Grove, PA). Sections were mounted in anti-fade medium containing 4′,6-Diamidin-2-phenylindol (DAPI) (Vector Laboratories).

### Histopathological analyses of optic nerves and retinae

For all histopathological investigations, a minimum of 10 sections taken throughout either the length of each optic nerve or the retina were quantified. The number of APP positive axons was quantified using a morphometric grid and calculated per mm^2^ of the optic nerve. Relative axonal densities were determined in optic nerves cross sections stained with Bielschowsky’s silver impregnation by point sampling using a 25-point Olympus eyepiece, as previously described^57^. The degree of axon reduction is given as the percentage of axon density compared to the average axon density in healthy optic nerves. The number of Brn3a positive cells was quantified using a morphometric grid and calculated per mm of the retina.

### Visual evoked potential (VEP) measurement

Two weeks prior to immunization, and again at day 21 of EAE, animals were anaesthetised i.p. with ketamine (120 mg/kg) and xylazine (10 mg/kg). Measurements of visual evoked potentials (VEPs) were performed using the UTAS Visual Diagnostic System (LKC Technologies, Gaithersburg, MD, USA). Mice were placed on a heated pad and the temperature maintained at 37 °C. Prior to recording, pupils were dilated with 0.5% atropine (Ursapharm, Saarbrücken, Germany) and the animal dark-adapted for 5 minutes. Needle-type electrodes were placed in the primary visual cortex, 3mm lateral to lambda. A reference electrode was placed subcutaneously in the neck and the ground electrode was placed subcutaneously in the tail. Animals were placed in the dome equipped with a LED wholefield stimulator. Flash stimuli were presented from a distance of 20 cm with 0 dB intensity and frequency of 2 Hz. 100 sweeps were averaged for each recording. During the recording process, desiccation of eyes was prevented through the use of Liquifilm^®^ O.K. eye drops (Allergan, Westport, Ireland). This recording procedure was performed on each eye separately, and again on day 21 of EAE. The signal amplitude (μV) and latency (ms) were calculated from the first negative (N1) to the second positive (P2) peak of the response using software provided by the UTAS Visual Diagnostic System.

## Additional Information

**How to cite this article:** Lanz, T. V. *et al*. Tryptophan-2,3-Dioxygenase (TDO) deficiency is associated with subclinical neuroprotection in a mouse model of multiple sclerosis. *Sci. Rep.* **7**, 41271; doi: 10.1038/srep41271 (2017).

**Publisher's note:** Springer Nature remains neutral with regard to jurisdictional claims in published maps and institutional affiliations.

## Supplementary Material

Supplementary Figures
