# Supplementary material for: Tryptophan-2,3-Dioxygenase (TDO) deficiency is associated with subclinical neuroprotection in a mouse model of multiple sclerosis
Source: Sci Rep. 2017 Jan 24;7:41271. doi: 10.1038/srep41271 (PMC5259766; doi:10.1038/srep41271)
Supplement: Supplementary Figures [file srep41271-s1.pdf]

Tryptophan-2,3-Dioxygenase (TDO) deficiency is associated with subclinical neuroprotection in a mouse model of multiple sclerosis

Tobias V. Lanz, Sarah K. Williams, Aleksandar Stojic, Simeon Iwantscheff, Jana K. Sonner, Carl Grabitz, Simon Becker, Laura-Inés Böhler, Soumya R. Mohapatra, Felix Sahm, Günter Küblbeck, Toshikazu Nakamura, Hiroshi Funakoshi, Christiane A. Opitz, Wolfgang Wick, Ricarda Diem, Michael Platten

Supplementary Figures

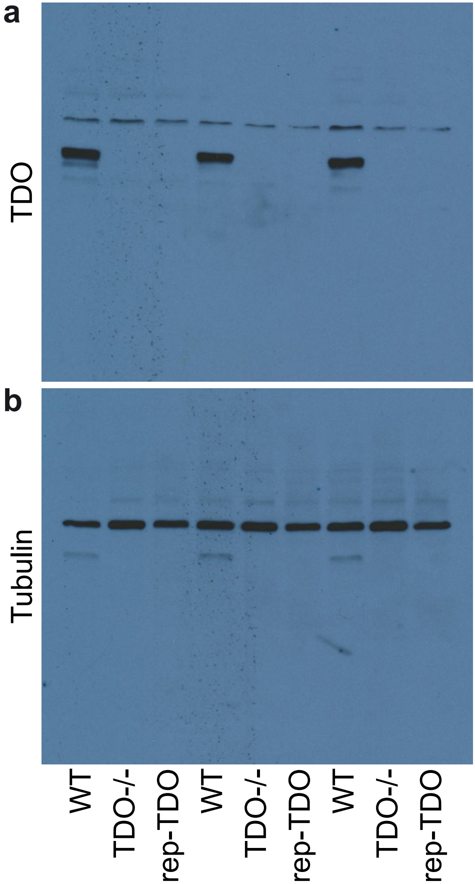

Supplementary Figure S1. TDO protein levels. Original western blot analyses, corresponding to the cropped western blots shown in Fig. 1c. Liver lysates were used from three different mice of each genotype as indicated. Raw images of the membrane stained for (a) TDO, and after stripping stained for (b) Tubulin.

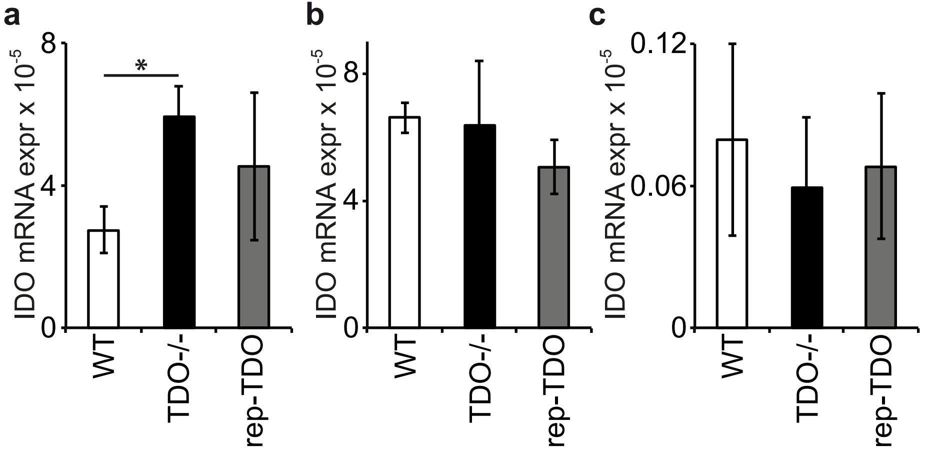

Supplementary Figure S2. IDO expression levels. qPCR analyses of (a) spleens, (b) lymph nodes and (c) livers of naive mice (WT and homozygous TDO-/- as well as reporter-TDO), measuring IDO mRNA expression levels in relation to GAPDH. Means ± SEM are shown from 3 mice per time point, each measured in duplicates and two serial dilutions. * $p < 0.05$ according to unpaired Student's t-test.

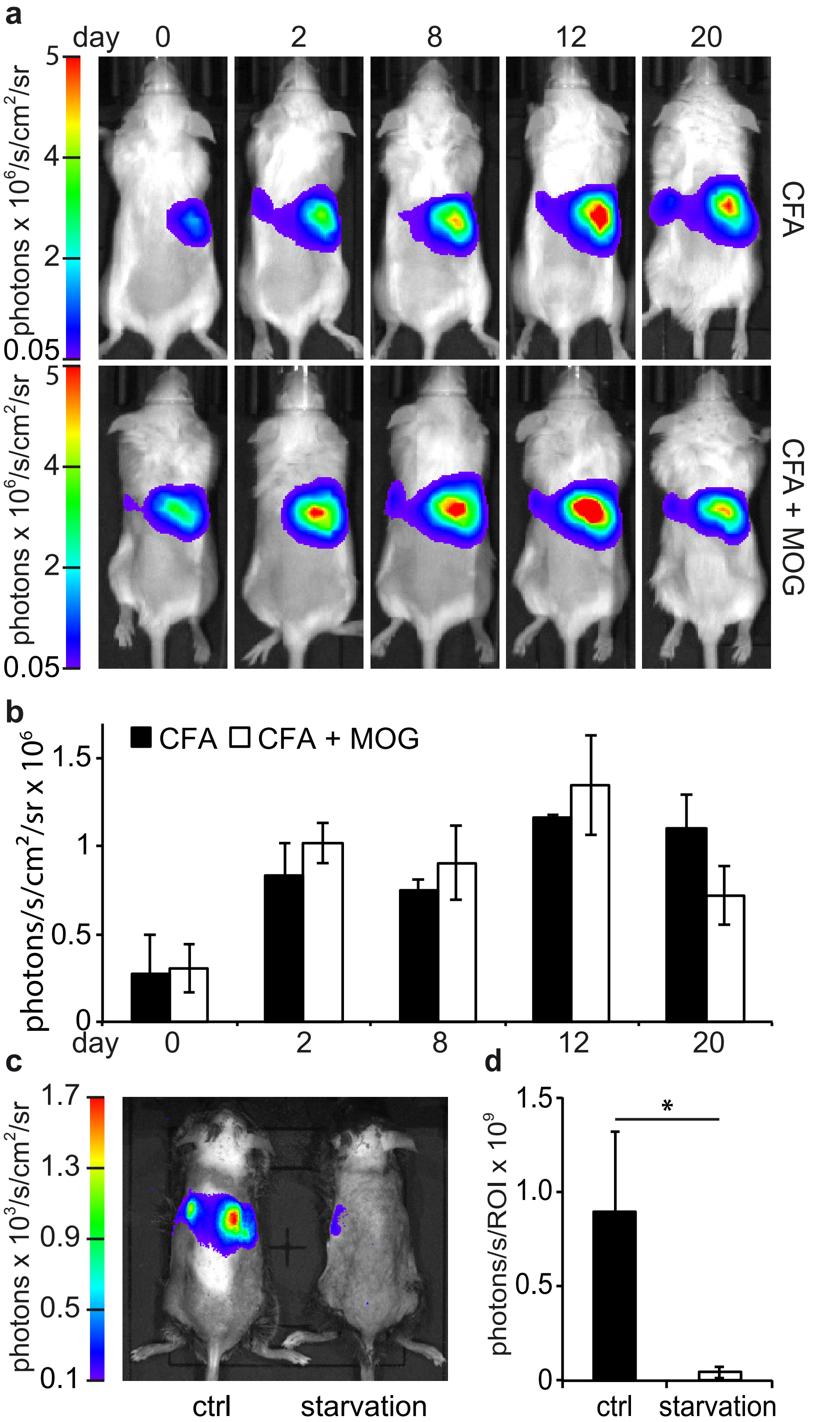

Supplementary Figure S3. TDO bioluminescence. (a) Luciferase imaging of two representative reporter-TDO mice, immunized with either CFA (upper row) or CFA + MOG35-55 (lower row) at indicated time points (days) after immunization. (b) Statistical evaluation of luciferase imaging, 3 mice per group. Means ± SEM are shown, no significant differences in bioluminescence between CFA and CFA + MOG group according to unpaired Student's t-test. (c) Luciferase imaging of two representative reporter-TDO mice, one with normal diet (left) and one after three days of starvation (right). (d) Statistical evaluation of luciferase imaging, 3 mice per group. Means ± SEM are shown. * $p < 0.05$ according to unpaired Student's t-test.

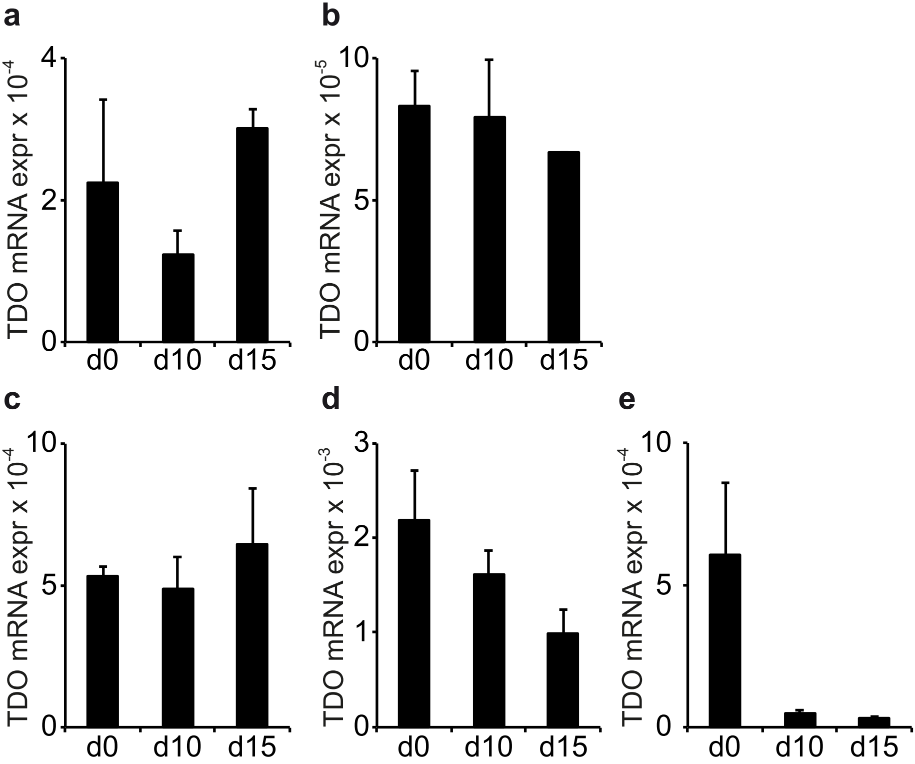

Supplementary Figure S4. TDO expression levels. (a-e) qPCR analyses of (a) spleens, (b) lymph nodes, (c) brains, (d) cerebella and (e) spinal cords, measuring TDO mRNA expression levels relative to GAPDH before immunization (d0) as well as 10 days (d10) and 15 days (d15) after immunization. Means ± SEM are shown from 4 mice per time point, each measured in triplicates. No significant differences in expression levels according to unpaired Student's t-test.

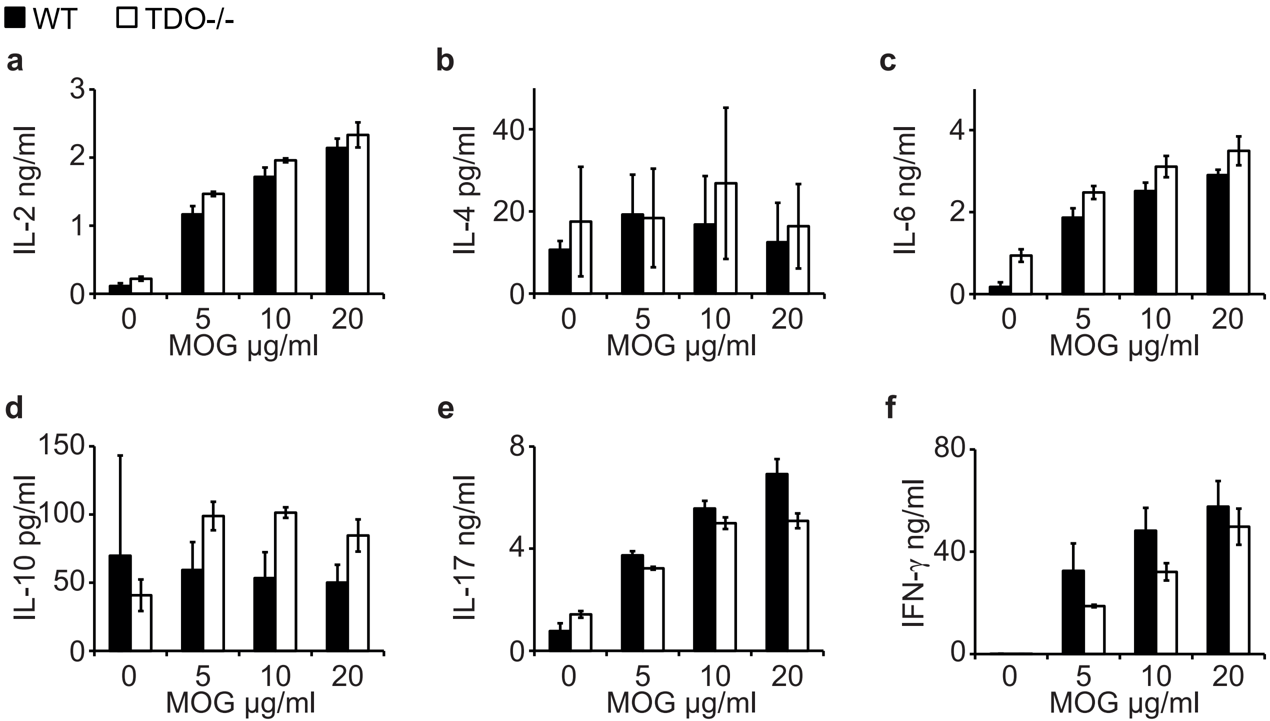

Supplementary Figure S5. Altered trp and kyn levels do not influence Th cell cytokine profiles. (a) – (f) Cytokine ELISAs of in-vitro T cell cultures from LN cells of immunized mice. (a) IL-2, (b) IL-4, (c) IL-6, (d) IL-10, (e) IL-17, (f) IFN-γ. LN cells were obtained from WT and TDO-/- mice on day 9 after immunization with MOG peptide 35-55 and cultured in 96-well plates with the indicated concentrations of MOG peptide 35-55. Supernatants were obtained for ELISA measurement 72h after onset of in-vitro culture (96h for IL-10). Each panel shows one representative experiment out of three independent experiments, each carried out in triplicates.
